# Association between nutrient patterns and hyperuricemia: mediation analysis involving obesity indicators in the NHANES

**DOI:** 10.1186/s12889-022-14357-5

**Published:** 2022-10-28

**Authors:** Juping Wang, Shuting Chen, Junkang Zhao, Jie Liang, Xue Gao, Qian Gao, Simin He, Tong Wang

**Affiliations:** 1 Department of Health Statistics, School of Public Health, Shanxi Medical University, No.56 Xinjian South Road, Taiyuan, 030001 China; 2 Department of Mathematics, School of Basic Medical Science, Shanxi Medical University, Taiyuan, China

**Keywords:** Obesity, Hyperuricemia, Mediation analysis, Principal component analysis, Reduced rank regression

## Abstract

**Background:**

Diet has long been hypothesized to play an important role in hyperuricemia, and weight gain is a factor that is strongly associated with the rise in serum urate. We aimed to clarify the mediating role of obesity in the relationship between diet and hyperuricemia and to determine whether a weight-loss diet is an effective way to prevent hyperuricemia.

**Methods:**

This cross-sectional study analysed representative samples of United States (*n* = 20,081; NHANES 2007–2016) adults. Nutrient patterns were derived with two methods: principal component analysis (PCA) and reduced rank regression (RRR) with obesity. Logistic regression and multivariable linear regression were applied to analyse the association between nutrient patterns in obesity and hyperuricemia. Mediation analyses were used to determine whether four obesity indicators, including body mass index (BMI), waist circumference (WC), visceral adiposity index (VAI) and lipid accumulation product index (LAP), mediated the relationship between nutrient patterns and hyperuricemia.

**Results:**

PCA revealed three nutrient patterns (including “Low energy diet”, “Lower vitamin A, C, K pattern” and “Vitamin B group”), and only Vitamin B group had a total effect on hyperuricemia. RRR revealed one main nutrient pattern associated with obesity, which was characterized by High fat and low vitamin levels and was significantly associated with hyperuricemia. Mediation analysis showed that obesity mostly or even completely mediated the relationship between nutrient patterns and hyperuricemia, especially traditional obesity indicators, which played a key intermediary effect. The proportions of indirect effects for BMI and WC were as high as 53.34 and 59.69, respectively.

**Conclusions:**

Our findings suggest that the direct effect of diet on hyperuricemia is weak, and obesity plays a critical mediating role in the relationship between diet and hyperuricemia, which confirms that a weight-loss diet such as a “Low fat and high vitamin diet” may be useful in preventing hyperuricemia.

**Supplementary Information:**

The online version contains supplementary material available at 10.1186/s12889-022-14357-5.

## Introduction

The latest Global Burden of Disease (GBD) showed that gout, the most common cause of inflammatory arthritis, affects 41 million people worldwide [1]. Hyperuricemia, as the early stage and major aetiologic factor of gout, needs to be given more attention. Hyperuricemia is caused by the elevation of plasma uric acid concentration in the human body and is defined as blood uric acid levels higher than 7.0 mg/dL (416 μmol/L) in men and 6.0 mg/dL (360 μmol/L) in women under normal dietary conditions [2, 3]. Hyperuricemia is also a potential risk factor for cardiovascular disease, type 2 diabetes, chronic kidney disease and mortality [4]. Recently, the prevalence of hyperuricemia has increased markedly worldwide, but management remains suboptimal [5].

As an important factor in many chronic diseases, diet is also hypothesized to be a contributing factor in hyperuricemia, and an increase in dietary purines leads to increased urate production [6]. According to the update on gout management, dietary modifications may be useful adjuncts to urate-lowering therapy [7]. Therefore, there has been much interest in the potential effects of dietary approaches in hyperuricemia management, and a large amount of literature has focused on evaluating the association between diet and hyperuricemia. For example, red meat, seafood, sugar-sweetened beverages, alcohol, and animal protein have been identified to be associated with a greater risk of hyperuricemia [8]. Many popular dietary patterns, such as the Med Diet Score [9], Dietary Approaches to Stop Hypertension (DASH) diet [10] and plant-based diets [11], have also been studied in relation to hyperuricemia.

On the other hand, obesity has also been hypothesized to be an important cause of elevated uric acid. For example, a longitudinal study of 2611 young adults reported that baseline BMI was positively related to a 10-year change in serum uric acid (UA) [12]. Bidirectional Mendelian randomization analyses showed that BMI was causally associated with elevated serum UA but not vice versa [13]. A randomized controlled trial found that bariatric surgery was associated with a significant urate reduction when compared with traditional therapy [14]. Another study also showed that bariatric surgery could reduce the incidence of gout, implying that obesity may be an important cause of gout [15].

Further, it is well known that dietary factors are important factors in obesity. Based on the above relationships among diet, obesity, and hyperuricemia, we naturally hypothesized that the relationship between diet and hyperuricemia may be mediated by obesity. Furthermore, we were interested in whether a weight-loss diet could have a preventive effect on hyperuricemia. In addition to body mass index (BMI) and waist circumference (WC), two other novel indicators of obesity, the visceral adiposity index (VAI) [16] and lipid accumulation product index (LAP), are also low-cost indicators and are often used to reflect obesity from different perspectives [17].

In addition, compared with a single dietary factor, dietary patterns have been widely used in nutritional research because they can reflect the overall dietary characteristics of individuals. Further, in an international research context, nutrients are universal and the nutrient patterns can be compared across varied ethnicities, so nutrient patterns may be more interpretable and much easier to translate into public health recommendations across populations [18], whereas dietary patterns may be affected by social, cultural and geographical scenarios [19]. Various approaches to dietary patterns were discussed in a review, and each method has a unique feature and serves a distinct purpose [20]. In addition to investigator-driven methods such as the Med Diet Score and Dietary Approaches to Stop Hypertension (DASH) diet, principal component analysis (PCA) and reduced rank regression (RRR) are also often used, where RRR is a hybrid method that combines a priori professional knowledge of health outcomes and the relevant relational structure of nutrients and is often used to complement data-driven methods [20].

Therefore, to further explore the relationship among nutrient patterns, obesity and hyperuricemia, the current study first identified the nutrient patterns based on two methods: principal component analysis and reduced rank regression with obesity. Furthermore, we aimed to examine the possible mediating role of multiple obesity indicators in the link between nutrient patterns and hyperuricemia by conducting mediation analyses.

## Methods

### Study populations

The National Health and Nutrition Survey (NHANES) is an ongoing continuous survey conducted by the Centers for Disease Control and Prevention (NCHS) to describe the health and nutritional status of the United States population [21]. Data are collected by using a complex, stratified, multistage probability cluster sampling design, and each survey cycle covers demographic data, body measurements, laboratory test results, and diet information [22]. The details of the programs, collection procedures and data files are publicly available at http://www.cdc.gov/nchs/nhanes.html. Participants in the NHANES provided written informed consent, and the study protocol was approved by the Research Ethics Review Board of the National Center for Health Statistics and the US Army Research Institute of Environmental Medicine Human Use Review Committee [23].

**Fig. 1:**
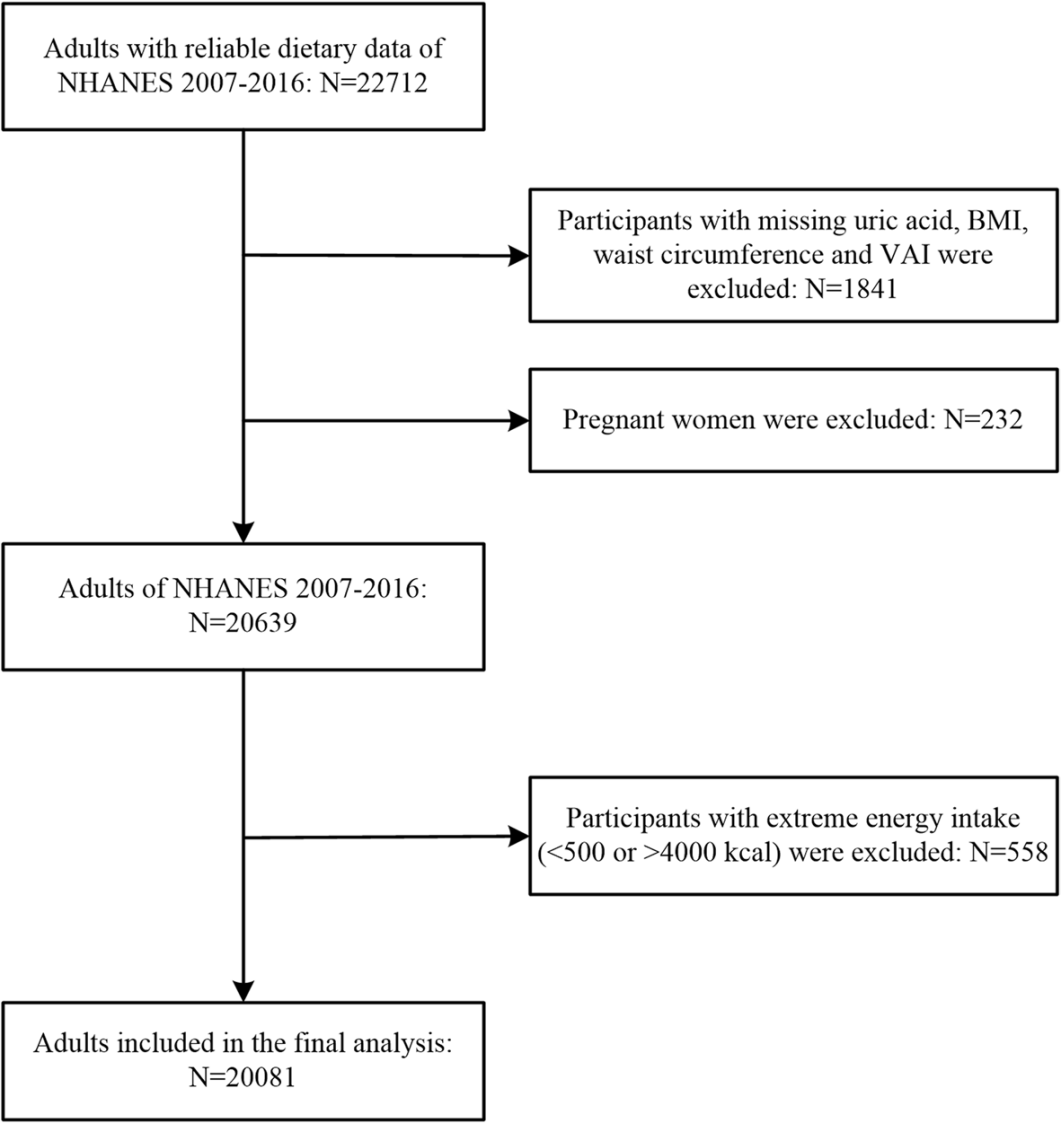
Flowchart showing the selection of the studied population

For this study, a total of 22,712 participants with reliable dietary NHANES data from 2007 to 2016 aged 20 years or older constituted the initial sample. After excluding pregnant women; individuals with missing uric acid, BMI, WC and VAI information; and those with extreme energy intake, 20,081 participants (9537 men and 10,544 women) were included in our final analyses (see Fig. 1).

### Dietary information

The dietary intake data were collected via two 24-h dietary recall interviews; the first dietary recall was collected with face-to-face inquiry, and the second dietary survey was conducted by telephone 3 to 10 days after the initial recall interview [22]. The food energy and nutrient contents of each food were calculated using the USDA Food and Nutrient Database for Dietary Studies [24]. We calculated the average intake of all nutrients from the two 24-h recalls. For simplicity, we did not take into account the specific saturated, monounsaturated and polyunsaturated fatty acids because we considered the sum of them. Finally, we considered 41 major nutrients.

### Assessment of mediators

Anthropometric and biochemical data were measured by NHANES researchers. WC was measured at the iliac crest by a tape measure to the nearest millimetre [22]. To assess the height and weight, participants wore their underwear, disposable paper robes and foam slippers [25]. BMI was calculated as weight in kilograms divided by the square of height in metres. A blood specimen was drawn from all study participants’ antecubital veins by a trained phlebotomist [25]. Laboratory testing details for haemoglobin A1c (HbA1c), direct HDL-cholesterol, and fasting triglycerides are provided in the NHANES Laboratory/Medical Technician Procedures Manual [22]. VAI was the integration of BMI, WC, TG and HDL: for males, $\textrm{VAI}=\left[\frac{\textrm{WC}\left[\textrm{cm}\right]}{39.68}+\left(1.88\times \textrm{BMI}\right)\right]\times \left(\frac{\textrm{TG}\left[\textrm{mmol}/\textrm{L}\right]}{1.03}\right)\times \left(\frac{1.31}{\textrm{HDL}\left[\textrm{mmol}/\textrm{L}\right]}\right);$ for females, $\textrm{VAI}=\left[\frac{\textrm{WC}\left[\textrm{cm}\right]}{36.58}+\left(1.89\times \textrm{BMI}\right)\right]\times \left(\frac{\textrm{TG}\left[\textrm{mmol}/\textrm{L}\right]}{0.81}\right)\times \left(\frac{1.52}{\textrm{HDL}\left[\textrm{mmol}/\textrm{L}\right]}\right)$[26]. LAP was the indicator used to evaluate lipid accumulation, and it combined WC and triglycerides (TGs): for males, LAP = (WC[cm] − 65) × TG[mmol/L]; for females, LAP = (WC[cm] − 58) × TG[mmol/L] [27].

### Serum uric acid measurement and hyperuricemia

Uric acid concentration was detected on a Beckman Synchron LX20 (Beckman Coulter, Inc., Brea, CA) using a colorimetric method [21]. Hyperuricemia was defined as uric acid ≥420 mmol/L in males and ≥ 360 mmol/L in females [28] or the use of uric acid-lowering drugs.

### Confounders

Based on the associations with nutrient patterns, hyperuricemia and obesity measures, the following factors were considered confounders: age (20–39, 40–59, > 59 years), sex (male, female), race (Mexican American, non-Hispanic white, non-Hispanic black, others), income status based on poverty index (0–1.3, 1.3–3.5, > 3.5) [29], smoking status (smoking at least 100 cigarettes in lifetime or not), drinking status (had at least 12 alcohol drinks/year or not), vigorous physical activity (yes or no), creatinine level and energy intake, and history of diseases (including diabetes, hypertension, cardiovascular diseases, cancer, liver disease and dyslipidaemia). Information on all of these confounders was obtained via standardized questionnaires or instrumental measurement. Hypertension was defined as a mean systolic blood pressure (SBP) ≥140 mmHg, a mean diastolic blood pressure (DBP) ≥90 mmHg, or a self-reported hypertension diagnosis [30]. Cardiovascular diseases were defined as a positive answer to the question “Have you ever been told you had congestive heart failure/coronary heart disease/angina/heart attack/stroke?” [31]. Dyslipidaemia was defined as the use of lipid-lowering medications or a low-density lipoprotein cholesterol level of ≥140 mg/dL, a high-density lipoprotein cholesterol level of < 40 mg/dL, or a triglyceride level of ≥150 mg/dL [32].

### Statistical analysis

We considered masked variance and used the weighting methodology in all analyses [33]. The survey package of R (version 4.0) was used to account for the complex sampling design [34]. The general characteristics of the participants were summarized and compared according to hyperuricemia status. All continuous variables are presented as the mean with standard deviation, and the categorical variables are presented as frequencies and percentages. Student’s t test (normally distributed data) or nonparametric test (nonnormally distributed data) was applied for continuous variables, and chi-squared tests were used for categorical variables.

Nutrient patterns were derived from 41 nutrients based on two main approaches: principal component analysis (PCA) and reduced rank regression (RRR) with obesity indicators as the response variable. PCA is a data-driven analysis, and the number of factors was decided based on eigenvalues, scree tests, and factor interpretability [35]. Nutrients with factor loadings ≥|0.2| were considered major contributors to the corresponding pattern and were retained. Orthogonal varimax rotation was applied to increase interpretability between the patterns. RRR was the second statistical approach used to derive nutrient patterns. For this method, patterns were identified based on a set of predefined response variables [36]. Four obesity indicators after log transformation were used as response variables, and we retained the main nutrient patterns in which coefficients of nutrients were below or above |0.15|. The number of nutrient patterns was determined by the number of response variables. Each participant obtained a factor score for each pattern, which indicated the degree of adherence to the specific pattern. As simple linear dose–response relationships are unlikely to be found in nutritional epidemiology, we classified participants based on the quartile of the factor scores [37]. In addition, we computed the mean of main nutrient intakes across categories of nutrient pattern scores and compared them using analysis of variance.

Both crude and adjusted weighted logistic regression models were used to investigate the association between the scores for each nutrient pattern derived by PCA and RRR with hyperuricemia: Model 1 unadjusted; Model 2 adjusted for age, sex, and race; and Model 3 additionally adjusted for smoking, drinking, vigorous physical activity, pox ratio, creatinine level, energy intake, history of diabetes, hypertension, cardiovascular diseases, cancer, liver disease and dyslipidaemia. In addition, we used multivariable-adjusted models to identify nutrient patterns associated with obesity indicators, and the lowest quartile was used as the reference group. Trend tests were also conducted. Finally, as suggested by VanderWeele [38], mediation analysis was performed to examine the potential mediating role of four obesity indicators on the relationship between nutrient patterns and hyperuricemia. Odds ratios (ORs) and 95% CIs for direct effects and indirect effects were calculated using the bootstrap method. The proportion of mediation was calculated using OR_DE_(OR_IE_ − 1)/(OR_DE_OR_IE_ − 1), where OR_DE_ is the OR for the direct effect and OR_IE_ is the OR for the indirect effect.

Sensitivity analyses were applied as the same steps above for mediation analysis in a new population that further excluded individuals who were taking uric acid-lowering drugs based on the above study populations and used continuous urate level as the outcome. All statistical analyses were conducted with SAS 9.4 and R 3.6.3. All tests were two-sided, and *P* < 0.05 was considered statistically significant.

## Results

### General characteristics of study participants

The baseline characteristics of the participants according to hyperuricemia status are shown in Table 1. Of the 20,081 study participants, 18.38% had hyperuricaemia. In general, participants with hyperuricemia were more likely to be older, male, and non-Hispanic black and have higher levels of obesity indicators (BMI, WC, VAI and LAP) and creatinine and lower levels of physical activity than those without hyperuricemia. In addition, a higher proportion of those classified as hyperuricemia smoked more, and a higher proportion of them suffered from other diseases.

**Table 1:** Baseline characteristics of participants according to hyperuricemia status

Characteristics	All participants (*n* = 20,081)		Hyperuricemia status				*P* value
			Yes (*N* = 3691)		No (*N* = 16,390)		
	Mean, n	SD, %	Mean, n	SD, %	Mean, n	SD, %	
*Age group, n (%)*							< 0.001
20 ~ 39	6396	31.8	889	24.1	5507	33.6	
40 ~ 59	6885	34.3	1099	29.8	5786	35.3	
60~	6800	33.9	1703	46.1	5097	31.1	
*Sex, n (%)*							< 0.001
Male	9537	47.5	1965	53.2	7572	46.2	
Female	10,544	52.5	1726	46.8	8818	53.8	
*Race, n (%)*							< 0.001
Hispanic	5186	25.8	681	18.5	4505	27.5	
Non-Hispanic white	3993	19.9	906	24.5	3087	18.8	
Non-Hispanic black	8940	44.5	1762	47.7	7178	43.8	
others	1962	9.8	342	9.3	1620	9.9	
*Poverty index, n (%)*							0.001
< 1.3	6336	31.5	1179	31.9	5157	31.5	
1.3 ~ 3.5	7504	37.4	1454	39.4	6050	36.9	
> =3.5	6241	31.1	1058	28.7	5183	31.6	
*Vigorous recreational activities, n (%)*							< 0.001
Yes	4435	22.1	610	16.5	3825	23.3	
No	15,646	77.9	3081	83.5	12,565	76.7	
*Smoking at least 100 cigarettes in lifetime, n (%)*							< 0.001
Yes	8840	44.0	1774	48.1	7066	43.1	
No	11,241	56.0	1917	51.9	9324	56.9	
*Hypertension, n (%)*							< 0.001
Yes	7491	37.3	1586	43.0	5386	32.9	
No	12,590	63.7	2105	57.0	11,004	67.1	
*Diabetes, n (%)*							< 0.001
Yes	2531	12.6	678	18.4	1853	11.3	
No	17,550	87.4	3013	81.6	14,537	88.7	
*Drinking, n (%)*							0.932
Yes	5637	28.1	1034	28.0	4603	28.1	
No	14,444	71.9	2657	72.0	11,787	71.9	
*CVD, n (%)*							< 0.001
Yes	2098	10.4	661	17.9	1437	8.8	
No	17,983	89.6	3030	82.1	14,953	91.2	
*Liver disease, n (%)*							< 0.001
Yes	768	3.8	186	5.0	582	3.6	
No	19,313	96.2	3505	95.0	15,808	96.4	
*Dyslipidaemia, n (%)*							< 0.001
Yes	7595	37.8	1848	50.1	5747	35.1	
No	12,486	62.2	1843	49.9	10,643	64.9	
*WC*^a^,* mean (SD)*	99.64	16.25	108.42	16.37	97.66	15.56	< 0.001
*BMI*^b^,* mean (SD)*	29.20	6.75	32.46	7.41	28.47	6.37	< 0.001
*VAI*^c^,* mean (SD)*	2.61	3.45	3.23	3.39	2.47	3.45	< 0.001
*LAP*^d^,* mean (SD)*	71.51	78.30	97.37	78.31	65.69	77.11	< 0.001
*Energery, mean (SD)*	1971.06	695.81	1924.74	703.04	1981.49	693.76	< 0.001
*creatinine, mean (SD)*	0.89	0.42	1.05	0.46	0.86	0.41	< 0.001

Continuous variables are presented as the mean and standard deviation (SD), and categorical variables are presented as counts and percentages

^a^WC means waist circumference

^b^BMI means body mass index

^c^VAI means visceral adiposity index

^d^LAP means lipid accumulation product index

In addition, in terms of demographics and health-related factors, there were no significant differences between the sample analysed in this study and the total NHANES (2007–2016) sample (Supplemental Table S1).

### Nutrient patterns

#### Principal component analysis

We derived 3 independent nutrient patterns based on the principal component analysis of a complex survey, which explained 71.6% of the total variance.

The first pattern was negatively correlated with protein, fat, carbohydrate, cholesterol, choline, sodium and selenium, therefore it was termed “Low energy intake”. The second pattern was negatively correlated with vitamin A, vitamin C, vitamin K, carotene, and lutein, therefore it was termed “Lower vitamin A, C, K pattern”. The third pattern was positively correlated with vitamin B6, B12, and folate, therefore it was termed “Vitamin B group”. The factor loadings for each nutrient pattern are shown in Supplemental Table S2.

#### Reduced rank regression

Only “High fat and low vitamin diet”was kept for further analyses based on RRR, since it explained the largest variance (20.01%) of the response variables. It was positively correlated with fat and cholesterol and a positive correlation with vitamin A, C, D, K, fibre and folate, therefore it was termed “High fat and low vitamin diet”. The factor loadings of the pattern and the correlation coefficients with the response variables are shown in Supplemental Table S3.

Supplemental Table S4 additionally showed the mean of main nutrient intakes across categories of nutrient pattern scores. The average intake of major nutrients showed a significant increase or decrease trend with the increase of the corresponding nutrient pattern scores (*p* < 0.001).

### Nutrient patterns and the risk of hyperuricemia and obesity

Multivariate logistic regression analyses of the associations between the 4 nutrient patterns and hyperuricemia are shown in Table 2. After adjusting for all confounders (Model 3), there were two patterns that were significantly related to hyperuricemia. Among them, “Vitamin B group” wasbased on principal component analysis, compared to the first quartile as a reference, and the ORs were 0.81 (0.67, 0.99), 0.75 (0.63, 0.89) and 0.63 (0.51, 0.77), respectively. In addition, “High fat and low vitamin diet”, based on RRR, was significantly related to hyperuricemia compared with the lowest quartile, and the adjusted OR indicated a dose-dependent relationship with each quartile increment (*P* for trend < 0.001). The OR in the highest quartile was 1.23 (1.06, 1.41).

**Table 2:** Odds ratios and 95% confidence intervals for the association between nutrient patterns and hyperuricemia

Nutrient patterns	Quartiles of nutrient pattern scores				*P*-trend
	Q1	Q2	Q3	Q4	
		*OR* (95% *CI*)	*OR* (95% *CI*)	*OR* (95% *CI*)	
**PCA**^a^					
Lower energy intake					
Model 1^c^	Ref.	0.79 (0.67, 0.94)	0.88 (0.76, 1.02)	0.88 (0.74, 1.05)	0.296
Model 2^d^	Ref.	0.85 (0.70, 1.02)	1.01 (0.85, 1.20)	1.04 (0.88, 1.24)	0.267
Model 3^e^	Ref.	0.84 (0.68, 1.03)	1.01 (0.78, 1.29)	1.00 (0.73, 1.37)	0.459
Low vitamin A, C, K pattern					
Model 1^c^	Ref.	1.07 (0.89, 1.29)	1.20 (1.01, 1.42)	1.25 (1.03, 1.52)	0.009
Model 2^d^	Ref.	1.04 (0.87, 1.25)	1.16 (0.97, 1.39)	1.22 (0.98, 1.52)	0.036
Model 3^e^	Ref.	1.01 (0.84, 1.22)	1.12 (0.93, 1.34)	1.17 (0.93, 1.46)	0.104
Vitamin B group					
Model 1^c^	Ref.	0.84 (0.70, 1.02)	0.74 (0.62, 0.89)	0.63 (0.51, 0.77)	< 0.001
Model 2^d^	Ref.	0.82 (0.67, 0.99)	0.74 (0.62, 0.88)	0.63 (0.51, 0.77)	< 0.001
Model 3^e^	Ref.	0.81 (0.67, 0.99)	0.75 (0.63, 0.89)	0.63 (0.51, 0.77)	< 0.001
**RRR**^b^					
High fat and low vitamin diet					
Model 1^c^	Ref.	1.09 (0.92, 1.29)	1.27 (1.10, 1.46)	1.37 (1.18, 1.58)	< 0.001
Model 2^d^	Ref.	1.11 (0.93, 1.32)	1.27 (1.09, 1.48)	1.32 (1.14, 1.52)	< 0.001
Model 3^e^	Ref.	1.00 (0.84, 1.19)	1.14 (0.97, 1.32)	1.23 (1.06, 1.41)	< 0.001

^a^PCA is the method of principal component analysis and included the “Lower energy intake”, “Low vitamin A, C, K pattern” and “Vitamin B group” nutrient patterns

^b^RRR stands for reduced rank regression and included the “High fat and low vitamin diet” pattern, which was related to obesity

^c^Model 1 was the crude model

^d^Model 2 was adjusted for age, race, and sex

^e^Model 3 was further adjusted for smoking, drinking, vigorous physical activity, pox ratio, creatinine level, energy intake, history of diabetes, hypertension, cardiovascular diseases, cancer, liver disease and dyslipidaemia

The results of multivariable linear regression analysis are shown in Table 3. All four nutrient patterns were correlated with BMI, WC and LAP (*P* for trend < 0.05). Furthermore, the VAI was also significantly correlated with “High fat and low vitamin diet”.

**Table 3:** The association between nutrient patterns and obesity indicators

Nutrient patterns	Quartiles of nutrient pattern scores				*P*-trend
	Q1	Q2 (95% *CI*)	Q3 (95% *CI*)	Q4 (95% *CI*)	
**PCA**^a^					
Lower energy intake					
BMI	0	−1.37 (−1.89, −0.84)	−1.93 (−2.59, −1.26)	−2.67 (−3.49, −1.85)	< 0.001
WC	0	−2.87 (−4.12, −1.63)	−4.51 (−5.99, −3.02)	−6.06 (−7.90, −4.23)	< 0.001
VAI	0	−0.22 (−0.41, −0.02)	−0.19 (−0.48, 0.09)	−0.28 (− 0.59, 0.04)	0.211
LAP	0	−8.89 (− 14.15, − 3.63)	− 11.02 (− 18.38, − 3.65)	−15.32 (− 24.25, −6.38)	0.003
Low vitamin A, C, K pattern					
BMI	0	0.29 (− 0.08, 0.66)	0.92 (0.55, 1.28)	1.02 (0.61, 1.42)	< 0.001
WC	0	0.95 (0.10, 1.80)	2.23 (1.38, 3.07)	2.77 (1.85, 3.70)	< 0.001
VAI	0	−0.11 (− 0.27, 0.05)	− 0.03 (− 0.19, 0.13)	0.11 (− 0.10, 0.31)	0.263
LAP	0	−0.74 (−4.35, 2.87)	2.42 (−1.13, 5.97)	3.70 (− 0.48, 7.88)	0.037
Vitamin B group					
BMI	0	−0.39 (− 0.86,0.07)	−0.95 (−1.40, − 0.51)	− 1.48 (− 1.92, − 1.05)	< 0.001
WC	0	−1.22 (−2.31, − 0.14)	− 2.35 (− 3.39, − 1.31)	−3.61 (− 4.59, − 2.63)	< 0.001
VAI	0	0.04 (−0.12, 0.19)	−0.07 (− 0.21, 0.08)	−0.10 (− 0.25, 0.05)	0.075
LAP	0	−0.89 (− 4.36, 2.58)	−4.05 (− 7.90, − 0.19)	− 6.47 (−9.90, − 3.04)	< 0.001
**RRR**^b^					
High fat and low vitamin diet					
BMI	0	0.91 (0.59, 1.24)	1.78 (1.41, 2.15)	2.73 (2.35, 3.11)	< 0.001
WC	0	2.44 (1.66, 3.23)	4.57 (3.68, 5.46)	6.89 (6.02, 7.77)	< 0.001
VAI	0	0.24 (0.07, 0.41)	0.28 (0.09, 0.47)	0.24 (0.07, 0.40)	0.005
LAP	0	7.87 (3.92, 11.83)	9.94 (5.51, 14.37)	13.61 (9.54, 17.68)	< 0.001

Linear regression models were used to estimate β and 95% CIs and adjusted for smoking, drinking, vigorous physical activity, pox ratio, creatinine level, energy intake, history of diabetes, hypertension, cardiovascular diseases, cancer, liver disease and dyslipidaemia

^a^PCA is the method of principal component analysis and included the “Lower energyintake”, “Low vitamin A, C, K pattern” and “Vitamin B group” nutrient patterns

^b^RRR stands for reduced rank regression and included the “High fat and low vitamin diet” pattern, which was related to obesity

### Mediating role of obesity indicators in the association between nutrient patterns and hyperuricemia

Table 4 presents the direct and indirect effects of nutrient patterns on hyperuricemia with obesity measures as mediators. Overall, all four obesity indicators mediated the relationship between each nutrient pattern and hyperuricemia. Furthermore, the direct effects of the other three nutrient patterns in relation to hyperuricemia were almost nonsignificant except for Vitamin B group. The findings suggest that the association of each nutrient pattern with hyperuricemia was mediated by obesity. Although the indirect and direct effects were in opposite directions for the two nutrient patterns and the proportion of indirect effects in this case could not be explained, we found that obesity measures (BMI, WC, LAP) fully mediated the relationship between “High fat and low vitamin diet”, based on RRR, and hyperuricemia. In particular, two common obesity measures (BMI and WC) had significant mediating effects on the relationships between all four nutrient patterns and hyperuricemia, and the mediating proportions were as high as 53.34 and 59.69, respectively. In addition, LAP also mediated the relationship between three nutrient patterns and hyperuricemia, although the indirect effect was not as large as that of BMI and WC.

**Table 4:** Mediating effects of obesity on the association between nutrient patterns and odds ratios of hyperuricemia

Nutrient patterns	Direct effects	Indirect effects	Proportion of indirect effect
	*OR* (95% *CI*)	*OR* (95% *CI*)	
**PCA**^a^			
Lower energy intake			
BMI	1.14 (1.00, 1.28)	0.94 (0.92, 0.95)	NA^c^
WC	1.13 (0.99, 1.28)	0.94 (0.92, 0.95)	NA^c^
LAP	1.06 (0.94, 1.21)	0.98 (0.97, 1.00)	NA^c^
VAI	1.05 (0.93, 1.08)	1.00 (0.99, 1.01)	0.12%
Low vitamin A, C, K pattern			
BMI	1.03 (0.96, 1.11)	1.03 (1.02, 1.04)	53.34%
WC	1.03 (0.96, 1.10)	1.04 (1.02, 1.05)	59.69%
LAP	1.05 (0.99, 1.12)	1.01 (1.00, 1.01)	18.54%
VAI	1.06 (0.99, 1.13)	1.00 (0.99, 1.01)	7.81%
Vitamin B group			
BMI	0.90 (0.84, 0.96)	0.95 (0.94, 0.97)	28.31%
WC	0.90 (0.83, 0.96)	0.95 (0.94, 0.97)	28.95%
LAP	0.87 (0.81, 0.93)	0.98 (0.97, 0.99)	8.49%
VAI	0.86 (0.81, 0.92)	0.99 (0.99, 1.00)	2.48%
**RRR**^b^			
High fat and low vitamin diet			
BMI	0.99 (0.94, 1.05)	1.08 (1.06, 1.09)	NA^c^
WC	0.98 (0.93, 1.04)	1.09 (1.07, 1.10)	NA^c^
LAP	1.07 (0.99, 1.11)	1.01 (1.01, 1.03)	27.77%
VAI	1.07 (1.01, 1.13)	1.01 (1.00, 1.01)	6.49%

Mediation analysis was adjusted for smoking, drinking, vigorous physical activity, pox ratio, creatinine level, energy intake, history of diabetes, hypertension, cardiovascular diseases, cancer, liver disease and dyslipidaemia

^a^PCA is the method of principal component analysis and included the “Lower energyintake”, “Low vitamin A, C, K pattern” and “Vitamin B group” nutrient patterns

^b^RRR stands for reduced rank regression and included the “High fat and low vitamin diet” pattern, which was related to obesity

^c^NA means the proportion of indirect effects could not be explained because the direction of the indirect and direct effects were opposite

### Sensitivity analyses

Sensitivity analysis showed similar results: “Vitamin B group” was negatively correlated with blood uric acid while “High fat and low vitamin pattern” were positively associated with urate level (Supplemental Table S5). Compared to the first quartile as reference, the subjects in the highest quartile of the Vitamin B group were associated with lower uric acid levels (*p* < 0.01), for the High fat and low vitamin pattern, the uric acid level of the highest quantile increased by 0.19 (0.13,0.25) compared with the lowest quantile. In addition, all four nutrient patterns were correlated with BMI, WC and LAP (Supplemental Table S6), which were also shown to be important mediators in the nutrient patterns and uric acid pathways (Supplemental Table S7).

## Discussion

In this study, we used both PCA and RRR to derive the nutrient patterns and explored their relationship with hyperuricemia and obesity. We found that Vitamin B group and the nutrient pattern related to weight loss had a significant total effect on hyperuricemia. Furthermore, the associations between all four nutrient patterns and hyperuricemia were mediated by obesity in a large proportion. The significant mediating effect of obesity combined with the significant total effect of hyperuricemia based on a reduced-rank regression suggests that a weight-loss diet may be an effective way to prevent elevated uric acid.

With principal component analysis, we obtained three nutrient patterns, and only Vitamin B group had a significant total effect on hyperuricemia after adjusting for all covariates. The relationship between Vitamin B group and urate level is still controversial, but there are some studies about individual B vitamins that may support our conclusions. For example, another NHANES study on individual B vitamins indicated that the intakes of folate and vitamin B12 were inversely related to the risk of HU in males, and only folate was found in females [39]. The findings from an in vitro and in vivo animal study showed that *Aster glehni* along with vitamin B6 might be used as functional nutrients in reducing serum uric acid levels in gout [40]. Another randomized controlled trial showed that uric acid was significantly decreased after 4 and 8 weeks of supplementation with vitamin B-12 and fish oil [41]. Furthermore, according to the results of a substudy of the China Stroke Primary Prevention Trial, compared with enalapril alone, the combination of enalapril and folic acid could reduce the magnitude of the increase in UA concentrations in hypertensive adults, which implied that high folic acid intake may be an adjuvant nutritional recommendation for preventing and treating hyperuricemia [42]. However, a Norwegian randomized controlled study in patients with coronary artery disease did not find any significant effect of folic acid and vitamin B-12 treatment on the risk of hyperuricemia [43]. According to the literature, this discrepancy could be due to important differences in the population characteristics and treatment regimen between the 2 studies [44]. Our study found that overall, Vitamin B group had a certain effect on hyperuricemia; however, more clinical trials are required for further verification and need to further clarify which B vitamins should be included and at what dose to maximize uric acid reduction.

Furthermore, the indirect effect of obesity was significant in the relationship of all four nutrient patterns to hyperuricemia, while the direct effect was small to none. This finding is consistent with recent studies on the relationship of diet to hyperuricemia by using the method of population attributable fractions (PAFs). In a Mendelian randomized study, the effects of four dietary patterns on hyperuricemia were fully mediated by BMI. In addition, the article also points out BMI produced PAFs for hyperuricemia of 59–69%, while diet had a relatively minor role in lowering uric acid in the three nongout cohorts [45]. Another study of the Third National Health and Nutrition Examination Survey reported that the corresponding PAFs of hyperuricemia cases for overweight or obese and nonadherence to a DASH-style diet were 44 and 8%, respectively [46]. All these findings supported our results that the association between diet and hyperuricaemia was mediated by obesity in a large proportion [47]. It is well known that diet plays a critical role in obesity and further has a subsequent risk of hyperuricemia, so the total effect of diet is greater than the PAF estimated for diet (direct effect and independent of BMI), as shown in previous studies [46, 48]. In addition, only the direct effect of vitamin B group on hyperuricemia was significant, which implied that other potential mechanisms existed in addition to obesity. Previous studies have shown several other potential mechanisms. According to reports, folic acid can effectively reduce total homocysteine, thereby reducing intracellular adenosyl-homocysteine, which may induce marked DNA damage and release purine nucleotides that result in the generation of UA [43].

To further illustrate that the relationship between diet and hyperuricemia is largely mediated by obesity, we performed reduced rank regression and obtained one main pattern associated with obesity, “High fat and low vitamin” diet, which had a significant promoting effect on hyperuricemia, and the effect was entirely caused by weight gain. In fact, a variety of studies show that greater BMI is associated with an increased risk of hyperuricemia and that weight loss is an important measure for the prevention and management of hyperuricemia and gout [46, 49, 50]. For example, a cohort study including 1189 patients observed over 6.45 years showed that obesity (BMI > 27) was an independent risk factor for incident gout among women with and without hyperuricaemia [51]. Furthermore, Mendelian randomization studies have found that obesity is causally associated with serum urate levels in the general population [52], a systematic review of randomized controlled trials found a significant reduction in serum uric acid levels following orlistat therapy in adults [50]. In addition, a meta-analysis including 20 cohort studies assessed the relationship of bariatric surgery with gout and serum urate, and the results showed that uric acid decreased by an average of 0.73 mg/dL 3 months after bariatric surgery and 1.91 mg/dL 3 years after surgery [53]. The 2020 ACR gout treatment guideline also recommends that people with hyperuricemic gout who are obese or overweight should use a weight loss program [49]. Furthermore, our research also found that VAI and LAP have certain direct and indirect effects, suggesting that metabolism-related obesity has an important effect on hyperuricemia, and we cannot ignore them. In conclusion, the results of the reduced rank regression further suggest that obesity is an important mediator in the pathway between dietary patterns and hyperuricemia. The significant total effect suggests that nutrient patterns for weight loss may be an effective way to prevent and treat hyperuricemia. This pattern is consistent with current recommended diets (e.g., Mediterranean, Dietary Approaches to Stop Hypertension (DASH), Nordic, vegan, vegetarian) that are associated with weight loss [54], and this pattern has a lower correlation with obesity-related chronic diseases [55, 56].

The present study has several strengths. First, we identified nutrient patterns based on two methods: principal component analysis and reduced rank regression with obesity. The two methods complement each other, and both suggest that obesity largely mediates the relationship between diet and hyperuricemia. Therefore, we further determined that one nutrient pattern for weight loss had a significant effect on the prevention of hyperuricemia. Second, a variety of obesity indicators (BMI, WC, VAI and LAP) were used to examine the potential mediating role of obesity in the relationship between nutrient patterns and hyperuricemia. Different obesity indicators can reflect multiple perspectives of obesity. In particular, VAI and LAP incorporate indicators such as sex and metabolism, which makes our analysis more comprehensive. Third, our analyses were based on a large sample population and used a complex sampling design, so the results can be extrapolated to the general population. Fourth, the patterns were based on nutrients rather than food groups, which makes it easier for nutritionists to provide public nutrition recommendations. Finally, we adjusted for many key confounding variables.

However, our study has some limitations that need to be noted. First, due to the observational design, a possible causal relationship cannot be established, and this needs to be addressed in future cohort studies. Second, we adjusted for many potential confounders, but we cannot completely rule out unmeasured confounders. Third, a more detailed analysis needs to be performed in future studies. For example, future studies should consider subdividing the population into two subgroups, those suffering from gout and those not suffering from gout, and then analyse the subgroups separately. In addition, each obesity index was used as the response variable to perform reduced rank regression to obtain a specific nutrient pattern and analyse its effect on hyperuricemia.

## Conclusion

In summary, this study explored the relationship among diet, various obesity indicators and hyperuricemia based on two methods. Studies have shown that obesity largely mediates the relationship between nutrient patterns and the risk of hyperuricemia. Our findings suggest that the direct effect of diet on hyperuricemia is weak, and obesity plays a critical mediating role in the pathway between diet and hyperuricemia, which confirms the findings of recent studies that dietary factors contribute far less to hyperuricemia than obesity because diet has little direct effect on uric acid. Furthermore, the significant mediating effect of obesity combined with the significant total effect of hyperuricemia, based on a reduced-rank regression, suggests that a weight-loss diet may be a useful adjunct to urate-lowering therapy.

## Supplementary Information

**Additional file 1: Table S1.** Comparison of the characteristics between the analytical sample and full NHANES (2007–2016) sample. **Table S2.** Factor loadings of three nutrient patterns based on principal component analysis. **Table S3.** Factor loadings of nutrient pattern based on RRR and Spearman’s correlation coefficient with four obesity measures. **Table S4.** Mean of nutrient intakes across quarters of nutrient pattern scores. **Table S5.** Multivariate regression analysis relating nutrient patterns to uric acid level. **Table S6.** The association between nutrient patterns and obesity indicators for sensitivity analysis. **Table S7.** Mediating effects of obesity on the association between nutrient patterns and urate level.

## Data Availability

The datasets generated and analysed during the current study are publicly available from the National Center for Health Statistics at https://wwwn.cdc.gov/nchs/nhanes/Default.aspx.

## Abbreviations

BMI: Body mass index
VAI: Visceral adiposity index
WC: Waist circumference
LAP: Lipid accumulation product index
NHANES: The National Health and Nutrition Examination Survey
OR: Odds ratio
PCA: Principal component analysis
RRR: Reduced rank regression
