# Supplementary material for: Association between nutrient patterns and hyperuricemia: mediation analysis involving obesity indicators in the NHANES
Source: BMC Public Health. 2022 Oct 28;22:1981. doi: 10.1186/s12889-022-14357-5 (PMC9617335; doi:10.1186/s12889-022-14357-5)
Supplement: Supplementary file 1 — Additional file 1: Table S1. Comparison of the characteristics between the analytical sample and full NHANES (2007–2016) sample. Table S2. Factor loadings of three nutrient patterns based on principal component analysis. Table S3. Factor loadings of nutrient pattern based on RRR and Spearman’s correlation coefficient with four obesity measures. Table S4. Mean of nutrient intakes across quarters of nutrient pattern scores. Table S5. Multivariate regression analysis relating nutrient patterns to uric acid level. Table S6. The association between nutrient patterns and obesity indicators for sensitivity analysis. Table S7. Mediating effects of obesity on the association between nutrient patterns and urate level. [file 12889_2022_14357_MOESM1_ESM.doc]

| **Table S1** Comparison of the characteristics between the analytical sample and full NHANES (2007-2016) sample. | | | | | | | |
| --- | --- | --- | --- | --- | --- | --- | --- |
|  |  |  |  |  |  |  |  |
|  |  |  |  |  |  | |  |
| Characteristics | Analytical sample (N = 20081) | |  | Total sample (N=22712) | | | P value |
|  |  |  |  |  |  |  |  |
|  | Mean, n | SD, % |  | Mean, n | | SD, % |  |
| *Age group, n (%)^a^* |  |  |  |  | |  | 0.159 |
| 20~39 | 6396 | 31.8 |  | 7397 | | 32.6 |  |
| 40~59 | 6885 | 34.3 |  | 7608 | | 33.5 |  |
| 60~ | 6800 | 33.9 |  | 7707 | | 33.9 |  |
| *Sex, n (%)* |  |  |  |  | |  | 0.754 |
| Male | 9537 | 47.5 |  | 10821 | | 47.6 |  |
| Female | 10544 | 52.5 |  | 11891 | | 52.4 |  |
| *Race, n(%)* |  |  |  |  | |  | 0.019 |
| Hispanic | 5186 | 25.8 |  | 5721 | | 25.2 |  |
| Non-Hispanic white | 3993 | 19.9 |  | 4791 | | 21.1 |  |
| Non-Hispanic black | 8940 | 44.5 |  | 9993 | | 44.0 |  |
| others | 1962 | 9.8 |  | 2207 | | 9.7 |  |
| *Poverty index, n(%)* |  |  |  |  | |  | 0.098 |
| <1.3 | 6336 | 31.6 |  | 7362 | | 32.4 |  |
| 1.3~3.5 | 7504 | 37.4 |  | 8470 | | 37.3 |  |
| >=3.5 | 6241 | 31.1 |  | 6880 | | 30.3 |  |
| *Vigorous recreational activities, n(%)* |  |  |  |  | |  | 0.391 |
| Yes | 4435 | 22.1 |  | 4938 | | 21.7 |  |
| No | 15646 | 77.9 |  | 17774 | | 78.3 |  |
| *Smoking at least 100 cigarettes in life, n(%)* |  |  |  |  | |  | 0.825 |
| Yes | 8840 | 44.0 |  | 10080 | | 44.4 |  |
| No | 11241 | 56.0 |  | 12632 | | 55.6 |  |
| *Hypertension, n(%)* |  |  |  |  | |  | 0.406 |
| Yes | 7491 | 37.3 |  | 8561 | | 37.7 |  |
| No | 12590 | 62.7 |  | 14151 | | 62.3 |  |
| *Diabetes, n (%)* |  |  |  |  | |  | 0.394 |
| Yes | 2531 | 12.6 |  | 2963 | | 13.0 |  |
| No | 17550 | 87.4 |  | 19749 | | 87.0 |  |
| *Cardiovascular disease，n(%)* |  |  |  |  | |  | 0.062 |
| *Yes* | 2098 | 10.4 |  | 2500 | | 11.0 |  |
| *No* | 17983 | 89.6 |  | 20212 | | 89.0 |  |
| *Liver disease, n(%)* |  |  |  |  | |  | 0.615 |
| *Yes* | 768 | 3.8 |  | 890 | | 3.9 |  |
| *No* | 19313 | 96.2 |  | 21822 | | 96.1 |  |
| *Cancer,n(%)* |  |  |  |  | |  | 0.425 |
| *Yes* | 1987 | 9.9 |  | 2300 | | 10.1 |  |
| *No* | 18094 | 90.1 |  | 20412 | | 89.9 |  |
| *Dyslipidaemia, n(%)* |  |  |  |  | |  | 0.837 |
| Yes | 7595 | 37.8 |  | 8612 | | 37.9 |  |
| No | 12486 | 62.2 |  | 14100 | | 62.1 |  |
| *Drinking, n(%)* |  |  |  |  | |  | 0.188 |
| Yes | 5637 | 28.1 |  | 6506 | | 28.6 |  |
| No | 14444 | 71.9 |  | 16206 | | 71.4 |  |
| *Waist circumference* | 99.64 | 16.25 |  | 99.84 | | 16.50 | 0.439 |
| *BMI* | 29.20 | 6.75 |  | 29.29 | | 6.90 | 0.598 |
| *VAI* | 2.61 | 3.45 |  | 2.62 | | 3.40 | 0.89 |
| *LAP* | 71.51 | 78.3 |  | 72.15 | | 79.30 | 0.495 |
| *Energery* | 1971.06 | 695.81 |  | 2014.44 | | 814.10 | 0.149 |
| *creatinine* | 0.90 | 0.42 |  | 0.91 | | 0.46 | 0.596 |

Continuous variables are expressed as mean and standard [deviation](javascript:;) (SD), categorical variables presented are as counts and percentage.

| **Table S2** Factor loadings of three nutrient patterns based on principal component analysis. | | | |
| --- | --- | --- | --- |
| **Nutrients** | Lower energy intake | Low vitamin A, C, K pattern | Vitamin B group |
| Total fat | -0.26 |  |  |
| Monounsaturated fatty acids | -0.26 |  |  |
| Saturated fatty acids | -0.25 |  |  |
| Polyunsaturated fatty acids | -0.23 |  |  |
| Cholesterol | -0.23 |  |  |
| Protein | -0.23 |  |  |
| choline | -0.23 |  |  |
| Sodium | -0.23 |  |  |
| Selenium | -0.22 |  |  |
| Phosphorus | -0.22 |  |  |
| Carbohydrate | -0.20 |  |  |
| Vitamin C |  | -0.21 |  |
| Vitamin A |  | -0.26 |  |
| Lutein + zeaxanthin |  | -0.42 |  |
| Vitamin K |  | -0.45 |  |
| Beta-carotene |  | -0.48 |  |
| Alpha-carotene |  | -0.41 |  |
| Added alpha-tocopherol |  |  | 0.34 |
| Vitamin B12 |  |  | 0.28 |
| Added vitamin B12 |  |  | 0.45 |
| Retinol |  |  | 0.25 |
| Folic acid |  |  | 0.34 |
| Folate |  |  | 0.25 |
| Total folate |  |  | 0.21 |
| Vitamin B6 |  |  | 0.22 |
| Potassium |  |  |  |
| Magnesium |  |  |  |
| Food folate |  |  |  |
| Suger |  |  |  |
| Calcium |  |  |  |
| Zinc |  |  |  |
| Niacin |  |  |  |
| Fiber |  |  |  |
| Vitamin B1 |  |  |  |
| Vitamin B2 |  |  |  |
| Copper |  |  |  |
| Vitamin E as alpha-tocopherol |  |  |  |
| Iron |  |  |  |
| Lycopene |  |  |  |
| Beta-cryptoxanthin |  |  |  |
| Vitamin D |  |  |  |
| **Percent of variance explained** | 0.58 | 0.078 | 0.058 |

| **Table S3** Factor loadings of nutrient pattern based on RRR  and Spearman’s correlation coefficient with four obesity measures. | | | |
| --- | --- | --- | --- |
|
| **Nutrients** | High fat and low vitamin diet |  |  |
| Cholesterol | 0.33 |  |  |
| Saturated fatty acids | 0.27 |  |  |
| Total fat | 0.25 |  |  |
| Monounsaturated fatty acids | 0.23 |  |  |
| Vitamin E as alpha-tocopherol | -0.15 |  |  |
| Vitamin D | -0.16 |  |  |
| Vitamin K | -0.16 |  |  |
| Alpha-carotene | -0.19 |  |  |
| Lutein + zeaxanthin | -0.19 |  |  |
| Folate | -0.21 |  |  |
| Vitamin C | -0.21 |  |  |
| Total folate | -0.23 |  |  |
| Fiber | -0.24 |  |  |
| Magnesium | -0.25 |  |  |
| Beta-carotene | -0.27 |  |  |
| Sodium |  |  |  |
| Polyunsaturated fatty acids |  |  |  |
| choline |  |  |  |
| Protein |  |  |  |
| Retinol |  |  |  |
| Vitamin B12 |  |  |  |
| Selenium |  |  |  |
| Phosphorus |  |  |  |
| Zinc |  |  |  |
| Food folate |  |  |  |
| Vitamin B2 |  |  |  |
| Niacin |  |  |  |
| Calcium |  |  |  |
| Suger |  |  |  |
| Lycopene |  |  |  |
| Iron |  |  |  |
| Beta-cryptoxanthin |  |  |  |
| Copper |  |  |  |
| Vitamin B1 |  |  |  |
| Carbohydrate |  |  |  |
| Vitamin A |  |  |  |
| Added alpha-tocopherol |  |  |  |
| Add vitamin B12 |  |  |  |
| Potassium |  |  |  |
| Vitamin B6 |  |  |  |
| Folic acid |  |  |  |
| **Percent of variance explained** | 0.20 |  |  |
| **Spearman’s correlation coefficient** |  |  |  |
| BMI | 0.61 |  |  |
| WC | 0.69 |  |  |
| VAI | 0.14 |  |  |
| LAP | 0.34 |  |  |

**Table S4** Mean of nutrient intakes across quarters of nutrient pattern scores

| Nutrient intakes | Quartiles of nutrient pattern scores | | | | *P*-trend |
| --- | --- | --- | --- | --- | --- |
|  | Q1 | Q2 | Q3 | Q4 |  |
| **PCA**^a^ |  |  |  |  |  |
| **Lower energy intake** |  |  |  |  |  |
| Total fat | 112.39 (28.88) | 80.40 (18.76） | 62.30 (15.64） | 41.65（14.06） | <0.001 |
| Monounsaturated fatty acids | 40.64 (11.95) | 28.78 (7.71) | 22.22 (6.36) | 14.75 (5.47) | <0.001 |
| Saturated fatty acids | 36.39 (11.68) | 25.87 (7.90) | 19.99 (6.54) | 13.38 (5.38) | <0.001 |
| Polyunsaturated fatty acids | 25.41 (9.08) | 18.53 (6.42) | 14.43 (5.22) | 9.64 (4.36) | <0.001 |
| Cholesterol | 422.82 (208.13) | 300.81 (142.55) | 233.22 (113.69) | 159.14 (92.47) | <0.001 |
| Protein | 115.64 (28.24) | 83.84 (16.08) | 66.90 (13.63) | 46.71 (13.23) | <0.001 |
| choline | 471.57 (148.47) | 343.81 (95.57) | 270.62 (75.93) | 186.83 (66.50) | <0.001 |
| Sodium | 4791.81(1223.62) | 3536.90 (806.07) | 2850.92 (675.22) | 2005.79(614.87) | <0.001 |
| Selenium | 160.65 (48.21) | 116.72 (29.00) | 93.21 (24.00) | 65.14 (21.46) | <0.001 |
| Phosphorus | 1917.97 (417.31) | 1390.30 (215.80) | 1105.55 (182.21) | 764.26 (195.18) | <0.001 |
| Carbohydrate | 333.13 (86.53) | 258.92 (65.56) | 214.65 (56.37) | 157.57 (50.33) | <0.001 |
| **Low vitamin A, C, K pattern** |  |  |  |  |  |
| Vitamin C | 137.94 (95.49) | 97.67 (64.08) | 66.23 (47.31) | 32.63 (29.52) | <0.001 |
| Vitamin A | 1027.79 (796.25) | 604.56 (317.34) | 455.81 (251.47) | 338.88 (219.26) | <0.001 |
| Lutein + zeaxanthin | 3887.94 (4968.26) | 1239.02 (801.75) | 746.51 (464.42) | 423.69 (298.86) | <0.001 |
| Vitamin K | 233.46 (345.05) | 96.76 (52.03) | 65.75 (34.75) | 43.14 (25.09) | <0.001 |
| Beta-carotene | 5839.46 (5556.42) | 1864.59 (1159.99) | 863.89 (577.76) | 371.60 (295.95) | <0.001 |
| Alpha-carotene | 1120.43 (1790.73) | 364.88 (385.55) | 150.12 (196.72) | 43.82 (78.95) | <0.001 |
| Vitamin B group |  |  |  |  |  |
| Added alpha-tocopherol | 0.01 (0.15) | 0.09 (0.50) | 0.33 (1.14) | 2.113 (4.85) | < 0.001 |
| Vitamin B12 | 2.43 (1.38) | 3.62 (1.78) | 4.81 (2.41) | 8.65 (7.46) | < 0.001 |
| Added vitamin B12 | 0.03 (0.18) | 0.16 (0.43) | 0.64 (0.92) | 3.02 (3.37) | < 0.001 |
| Retinol | 190.79 (115.85) | 298.24 (156.08) | 404.02 (197.12) | 705.36 (646.75) | < 0.001 |
| Folic acid | 74.87 (40.13) | 120.89 (53.65) | 174.63 (76.04) | 333.55 (193.04) | < 0.001 |
| Folate | 264.91 (85.47) | 406.36 (99.97) | 534.73 (140.27) | 838.53 (344.39) | < 0.001 |
| Total folate | 212.56 (67.87) | 321.78 (80.21) | 412.49 (109.10) | 605.00 (228.78) | < 0.001 |
| Vitamin B6 | 1.18 (0.46) | 1.64 (0.52) | 2.06 (0.67) | 3.15 (1.67) | < 0.001 |
| **RRR^b^** |  |  |  |  |  |
| **High fat and low vitamin diet** |  |  |  |  |  |
| Cholesterol | 212.27 (135.99) | 227.63 (133.71) | 272.32 (144.74) | 403.81 (206.67) | < 0.001 |
| Saturated fatty acids | 20.16 (10.52) | 20.81 (10.27) | 23.64 (10.73) | 31.01 (12.28) | < 0.001 |
| Total fat | 65.85 (30.33) | 64.30 (28.86) | 72.20 (29.57) | 94.39 (33.46) | < 0.001 |
| Monounsaturated fatty acids | 23.68 (12.02) | 22.82 (10.93) | 25.80 (11.20) | 34.08 (12.85) | < 0.001 |
| Vitamin E as alpha-tocopherol | 9.68 (6.81) | 7.05 (4.29) | 6.86 (3.92) | 7.60 (3.72) | < 0.001 |
| Vitamin D | 6.13 (6.07) | 4.33 (3.68) | 3.75 (3.12) | 4.07 (3.11) | < 0.001 |
| Vitamin K | 154.69 (319.53) | 117.74 (136.85) | 98.72 (115.45) | 67.96 (85.74) | <0.01 |
| Alpha-carotene | 724.92 (1717.27) | 402.00 (668.81) | 299.73 (578.23) | 252.68 (492.02) | < 0.001 |
| Lutein + zeaxanthin | 2591.53 (4662.47) | 1450.93 (2318.41) | 1132.86 (1569.48) | 1122.10(1443.0) | < 0.001 |
| Folate | 638.77 (358.18) | 486.86 (264.82) | 449.57 (238.50) | 469.26 (239.19) | < 0.001 |
| Vitamin C | 113.43 (89.64) | 83.48 (70.36) | 72.29 (64.11) | 65.27 (62.73) | < 0.001 |
| Total folate | 487.16 (242.07) | 368.26 (179.12) | 339.83 (163.31) | 356.51 (165.08) | < 0.001 |
| Fiber | 21.16 (10.25) | 16.10 (7.86) | 14.61 (7.26) | 14.79 (7.32) | < 0.001 |
| Magnesium | 353.48 (137.21) | 270.93 (104.33) | 253.18 (100.38) | 267.78 (99.41) | < 0.001 |
| Beta-carotene | 3946.91 (5831.64) | 2089.98 (2518.08) | 1537.01 (1916.38) | 1366.04(1706.2) | < 0.001 |

^a^ PCA is the method of principal component analysis and included the “Lower energy intake”, “Low vitamin A, C, K pattern” and “Vitamin B group” nutrient patterns.

^b^ RRR stands for reduced rank regression and included the “High fat and low vitamin diet” pattern which is related to obesity.

**Table S5** Multivariate regression analysis relating nutrient patterns to uric acid level

| Nutrient patterns | Quartiles of nutrient pattern scores | | | | *P*-trend |
| --- | --- | --- | --- | --- | --- |
|  | Q1 | Q2 | Q3 | Q4 |  |
| **PCA**^a^ |  |  |  |  |  |
| Lower energy intake |  |  |  |  |  |
| Model 1 ^c^ | 0 | -0.32 (-0.41,-0.23) | -0.51 (-0.58,-0.43) | -0.66 (-0.76,-0.55) | <0.001 |
| Model 2 ^d^ | 0 | -0.03 (-0.11,0.05) | -0.02 (-0.1,0.06) | 0.02(-0.07,0.11) | 0.767 |
| Model 3 ^e^ | 0 | -0.02 (-0.10,0.06) | -0.01 (-0.10,0.09) | 0.00(-0.12,0.12) | 0.861 |
| Low vitamin A, C, K pattern |  |  |  |  |  |
| Model 1 ^c^ | 0 | 0.08 (-0.01,0.17) | 0.21 (0.13,0.29) | 0.28 (0.19,0.38) | <0.001 |
| Model 2 ^d^ | 0 | 0.04 (-0.04,0.12) | 0.12 (0.05,0.20) | 0.14 (0.05,0.23) | <0.001 |
| Model 3 ^e^ | 0 | 0.01 (-0.07,0.08) | 0.07 (0.01,0.14) | 0.07 (-0.01,0.16) | 0.048 |
| Vitamin B group |  |  |  |  |  |
| Model 1 ^c^ | 0 | -0.06 (-0.15,0.03) | -0.23 (-0.33,-0.13) | -0.26 (-0.37,-0.16) | <0.001 |
| Model 2 ^d^ | 0 | -0.05 (-0.13,0.03) | -0.2 (-0.29,-0.12) | -0.28 (-0.37,-0.19) | <0.001 |
| Model 3 ^e^ | 0 | -0.06 (-0.14,0.02) | -0.19 (-0.27,-0.11) | -0.26 (-0.34,-0.17) | <0.001 |
| **RRR**^b^ |  |  |  |  |  |
| High fat and low vitamin diet |  |  |  |  |  |
| Model 1 ^c^ | 0 | 0.04(-0.05,0.13) | 0.11 (0.03,0.19) | 0.34 (0.26, 0.43) | <0.001 |
| Model 2 ^d^ | 0 | 0.14 (0.06,0.21) | 0.18 (0.11,0.25) | 0.24 (0.17,0.30) | <0.001 |
| Model 3 ^e^ | 0 | 0.07 (0.00,0.14) | 0.11 (0.04,0.18) | 0.19 (0.13,0.25) | <0.001 |

^a^ PCA is the method of principal component analysis and included the “Lower energy intake”, “Low vitamin A, C, K pattern” and “Vitamin B group” nutrient patterns.

^b^ RRR stands for reduced rank regression and included the “High fat and low vitamin diet” pattern which is related to obesity.

^c^ Model 1 was the crude model;

^d^ Model 2 was adjusted for age, race, sex;

^e^ Model 3 was further adjusted for smoking, drinking, vigorous physical activity, pox ratio, creatinine level, energy intake, history of diabetes, hypertension, cardiovascular diseases, cancer, liver disease and dyslipidaemia.

.

**Table S6** The association between nutrient patterns and obesity indicators for sensitivity analysis

| Nutrient patterns | Quartiles of nutrient pattern scores | | | | *P*-trend |
| --- | --- | --- | --- | --- | --- |
|  | Q1 | Q2 | Q3 | Q4 |  |
| **PCA**^a^ |  |  |  |  |  |
| Lower energy intake |  |  |  |  |  |
| BMI | 0 | -1.37 (-1.9,-0.85) | -1.92 (-2.59,-1.25) | -2.65 (-3.47,-1.82) | <0.001 |
| WC | 0 | -2.89 (-4.12,-1.65) | -4.47 (-5.97,-2.98) | -5.99 (-7.83,-4.15) | <0.001 |
| VAI | 0 | -0.22 (-0.42,-0.02) | -0.20 (-0.48,0.09) | -0.27 (-0.59,0.04) | 0.209 |
| LAP | 0 | -8.94 (-14.23,-3.64) | -10.99 (-18.33,-3.65) | -15.18 (-24.06,-6.31) | 0.003 |
| Low vitamin A, C, K pattern |  |  |  |  |  |
| BMI | 0 | 0.29 (-0.09,0.66) | 0.92 (0.55,1.28) | 1.01 (0.60,1.41) | <0.001 |
| WC | 0 | 0.93 (0.08,1.79) | 2.23 (1.38,3.07) | 2.75 (1.82,3.68) | <0.001 |
| VAI | 0 | -0.11 (-0.27,0.05) | -0.03 (-0.19,0.13) | 0.10 (-0.10,0.31) | 0.274 |
| LAP | 0 | -0.77 (-4.40,2.86) | 2.41 (-1.18,6.00) | 3.60 (-0.56,7.76) | 0.040 |
| Vitamin B group |  |  |  |  |  |
| BMI | 0 | -0.38 (-0.83,0.08) | -0.93 (-1.37,-0.49) | -1.46 (-1.89,-1.03) | <0.001 |
| WC | 0 | -1.17 (-2.25,-0.08) | -2.29 (-3.32,-1.27) | -3.55 (-4.52,-2.58) | <0.001 |
| VAI | 0 | 0.04 (-0.12,0.19) | -0.07 (-0.21,0.08) | -0.10 (-0.25,0.05) | 0.076 |
| LAP | 0 | -0.80 (-4.30,2.70) | -3.92 (-7.79,-0.05) | -6.36 (-9.85,-2.88) | <0.001 |
| **RRR**^b^ |  |  |  |  |  |
| High fat and low vitamin diet |  |  |  |  |  |
| BMI | 0 | 0.79 (0.47,1.10) | 1.69 (1.35,1.10) | 2.68 (2.31,3.05) | <0.001 |
| WC | 0 | 2.10 (1.36,2.85) | 4.35 (3.49, 5.20) | 6.76 (5.93,7.59) | <0.001 |
| VAI | 0 | 0.06 (-0.06,1.96) | 0.17 (0.02,0.31) | 0.17 (0.03,0.31) | 0.007 |
| LAP | 0 | 3.37 (0.49,6.26) | 6.99 (3.73,10.24) | 11.78 (8.45,15.11) | <0.001 |

Linear regression models were used to estimate β and 95% CIs and adjusted for smoking, drinking, vigorous physical activity, pox ratio, creatinine level, energy intake, history of diabetes, hypertension, cardiovascular diseases, cancer, liver disease and dyslipidaemia..

^a^ PCA is the method of principal component analysis and included the “Lower energy intake”, “Low vitamin A, C, K pattern” and “Vitamin B group” nutrient patterns.

^b^ RRR stands for reduced rank regression and included the “High fat and low vitamin diet” pattern which is related to obesity.

**Table S7** Mediating effects of obesity on the association between nutrient patterns and urate level.

| Nutrient patterns | Direct effects ^a^ | Indirect effects ^a^ | Proportion of indirect effect |
| --- | --- | --- | --- |
| **PCA**^a^ |  |  |  |
| Lower energy intake |  |  |  |
| BMI | 0.048 (0.019, 0.084) | -0.045 (-0.059, -0.033) | NA ^c^ |
| WC | 0.047 (0.003, 0.083) | -0.044 (-0.056, -0.031) | NA ^c^ |
| LAP | 0.015 (-0.015, 0.053) | -0.012 (-0.019, -0.006 ) | NA ^c^ |
| VAI | 0.005 (-0.025, 0.043) | -0.002 (-0.005, 0.001 ) | NA ^c^ |
| Low vitamin A, C, K pattern |  |  |  |
| BMI | 0.007(-0.012, 0.028 ) | 0.020 ( 0.013, 0.028 ) | 73.76% |
| WC | 0.005(-0.016, 0.028) | 0.022( 0.016, 0.029 ) | 82.93% |
| LAP | 0.023( 0.001, 0.048 ) | 0.003( 0.000, 0.008 ) | 13.04% |
| VAI | 0.026 ( 0.004,0.051 ) | 0.001(-0.001, 0.003 ) | 3.31% |
| Vitamin B group |  |  |  |
| BMI | -0.062(-0.089,-0.032 ) | -0.028 (-0.038, -0.021 ) | 30.95% |
| WC | -0.062(-0.089,-0.033 ) | -0.028((-0.039, -0.019) | 30.84% |
| LAP | -0.084((-0.111,-0.052) | -0.006(-0.012,-0.002) | 6.83% |
| VAI | -0.089((-0.117,-0.057) | -0.001(-0.003,0.000) | 1.32% |
| **RRR**^b^ |  |  |  |
| High fat and low vitamin diet |  |  |  |
| BMI | 0.009 (-0.011，0.034) | 0.051(0.044,0.057) | 84.45% |
| WC | 0.006 (-0.014,0.029) | 0.054 (0.048,0.061) | 89.92% |
| LAP | 0.049 (0.029,0.078) | 0.011 (0.007,0.016) | 17.52% |
| VAI | 0.058 (0.037,0.086) | 0.002 (0.000,0.004) | 2.78% |

Mediation analysis was adjusted for smoking, drinking, vigorous physical activity, pox ratio, creatinine level, energy intake, history of diabetes, hypertension, cardiovascular diseases, cancer, liver disease and dys lipidaemia.

^a^ PCA is the method of principal component analysis and included the “Lower energy intake”, “Low vitamin A, C, K pattern” and “Vitamin B group” nutrient patterns.

^b^ RRR stands for reduced rank regression and included the “High fat and low vitamin diet” pattern which is related to obesity.

^c^ NA means the proportion of indirect effects could not be explained because the direction of indirect and direct effect is opposite.
